# Perfluorodecalin-based oxygenated emulsion as a topical treatment for chemical burn to the eye

**DOI:** 10.1038/s41467-022-35241-1

**Published:** 2022-11-30

**Authors:** Sanming Li, Kunpeng Pang, Shuyan Zhu, Kathryn Pate, Jia Yin

**Affiliations:** 1 Schepens Eye Research Institute of Massachusetts Eye and Ear, Harvard Medical School, Department of Ophthalmology, Boston, MA USA; 2 Coruna Medical, LLC, Longmont, CO USA

**Keywords:** Biomedical materials, Corneal diseases

## Abstract

Chemical injuries to the eye are emergencies with limited acute treatment options other than prompt irrigation and can cause permanent vision loss. We developed a perfluorodecalin-based supersaturated oxygen emulsion (SSOE) to topically deliver high concentration of oxygen to the eye. SSOE is manufactured in hyperbaric conditions and stored in a ready-to-use canister. Upon dispensation, SSOE rapidly raises partial oxygen pressure 3 times over atmospheric level. SSOE is biocompatible with human corneal cells and safe on mouse eyes in vivo. A single topical application of SSOE to the eye after alkali injury significantly promotes corneal epithelial wound healing, decreases anterior chamber exudation, and reduces optical opacity and cataract formation in mice. SSOE treatment reduces intraocular hypoxia, cell death, leukocyte infiltration, production of inflammatory mediators, and hypoxia-inducible factor 1-alpha signaling, thus hastening recovery of normal tissue integrity during the wound healing process. Here, we show that SSOE is an effective topical therapeutic in the acute treatment of ocular chemical injuries.

## Abstract (web summary)

Chemical injuries to the eye are medical emergencies with limited acute treatment options and can cause permanent vision loss. Here, the authors show that perfluorodecalin-based supersaturated oxygen emulsion is a safe and effective topical therapeutic in treating acute ocular chemical burn by reducing tissue hypoxia and inflammation.

## Introduction

Chemical burns, which represent up to 20% of eye injuries, occur in household, industrial, and military settings and are considered true ocular emergency that requires immediate evaluation and treatment^1,2^. The impact of severe chemical burns on the eye is profound, including eyelid skin deformity, corneal opacification and neovascularization, anterior chamber inflammation and exudation (hypopyon), cataract formation, and even damage to the retina and optic nerve^3^. Current treatments focus on the rapid removal of chemicals via irrigation and reducing subsequent inflammation. Despite these, detrimental damages to the eye still occur, resulting in permanent vision loss and blindness^4^.

The critical role of oxygen in maintaining tissue viability has long been recognized; and therapies to deliver oxygen to hypoxic tissue, such as the use of hyperbaric oxygen therapy in healing diabetic wounds, are now routine care^5^. Tissue hypoxia after the chemical burn has been noted in the eye and elsewhere^6^. Indeed, systemic delivery of oxygen, via nasal cannula or face mask, has been demonstrated to improve outcomes after chemical injury to the eye in animals and humans^7,8^. But the therapeutic benefits of these therapies are modest, and they require special equipment and personnel, and therefore infeasible outside well-equipped medical facilities or in delayed care.

Perfluorocarbons (PFCs) are inert chemicals with great biocompatibility and oxygen-dissolving capacity^9,10^. At room temperature, the solubility of oxygen (O_2_) is 40% or more in PFC, compared to 2.5% in water, 2.5% in plasma, and 20% in whole blood^11^. The high gas (including oxygen)-binding capacity of PFCs derives from fluorine’s low polarizability, and gas solubility in PFCs is directly proportional to the gas’s partial pressure^12^. Perfluorodecalin (PFD), a member of the PFC family, has been used as an oxygen carrier^13,14^ as well as an intraoperative adjuvant in ophthalmic surgery to reposition detached retina^15^. PFD-based oxygen emulsion has been shown to improve wound healing in second-degree burns in human and porcine skins^16,17^. Herein, we report the development of an ophthalmic PFD-based supersaturated oxygen emulsion (SSOE) and evaluate its safety and efficacy in mitigating acute ocular injury in a mouse model of alkali burn.

## Results

### SSOE is formulated with perfluorodecalin for ophthalmic use

**Fig. 1:**
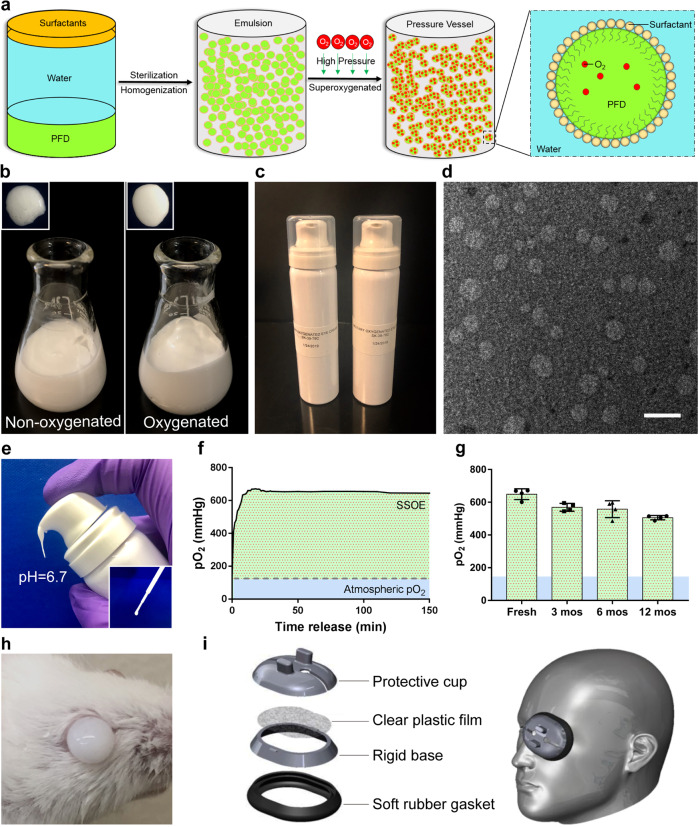
Formulation and characterization of ophthalmic supersaturated oxygen emulsion (SSOE). **a** Schematic depicting SSOE manufacture. PFD, surfactants and water are homogenized, and the emulsion is saturated with medical-grade oxygen in a high-pressure reaction vessel. Insert shows schematic depicting oxygen-carrying nanoparticles within the emulsion. **b** Compared with non-oxygenated emulsion, the oxygenated emulsion has a more uniform and reflective appearance. **c** SSOE is packaged into small pressurized canisters. **d** Electron microscopic image of SSOE nanoparticles (scale bar represents 200 nm). Data are representative of three independent experiments. **e** SSOE is readily dispensed from the canister and has a pH of 6.7 and can be transferred with a plastic pipette (insert). **f** Partial pressure of oxygen (pO2) with SSOE is above 600 mmHg upon dispensation for at least 2.5 h (dashed line: atmospheric pO2 of 160 mmHg). **g** SSOE maintains oxygen-releasing capacity for at least 1 year. Data are presented as mean ± SEM. *n* = 4 individual canisters at each timepoint. **h** SSOE remains on the mouse ocular surface after application. **i** An accessory device is designed for SSOE application in humans. Source data are provided as a Source Data file.

Of all PFCs, perfluorodecalin (PFD) has been most widely used in medical applications due to its high oxygen-carrying capacity, dissolving 49 mL of oxygen per 100 mL of PFD at room temperature and pressure^12^. PFCs including PFD are hydrophobic thus must be emulsified to promote water solubility. We used two surfactants, Phospholipon 90H and Polawax (fixed proportion 5.0:1.8), to emulsify PFD. The schematic in Fig. 1a depicts the formulation of supersaturated oxygen emulsion (SSOE). Briefly, 25% (w/v) PFD was homogenized with Phospholipon 90H, Polawax, and water; the emulsified nanoparticles were then supersaturated with medical-grade oxygen gas in a high-pressure reaction vessel. The oxygenated emulsion has a more uniform and reflective appearance, compared with the non-oxygenated emulsion (Fig. 1b). The final SSOE was packaged in small pressurized dispensing canisters (Fig. 1c). The structure of SSOE particles was observed under transmission electron microscopy (Fig. 1d). The diameter of most particles was between 50 and 300 nm.

The emulsion can be directly dispensed from the canister without additional equipment or manipulation (Fig. 1e). In addition, disposable pipettes can be used to transfer the emulsion after dispensation (Fig. 1e, inset). The pH of the emulsion is 6.7, within the safety range of ophthalmic use^18^. Upon dispensation, the partial pressure of oxygen (pO_2_) within the emulsion reached the peak of 600 mmHg in a few minutes and sustained above 600 mmHg for at least 2.5 h (Fig. 1f), four times of the atmospheric pO_2_ (160 mmHg). The un-opened packaged emulsion was stored in normal environment at room temperature up to 1 year. pO_2_ of stored emulsion decreased slightly compared to freshly prepared emulsion, but still maintained above 500 mmHg when dispensed after 1 year of storage (Fig. 1g). Due to its inherent viscosity, SSOE remained on the ocular surface after application, as shown on a mouse eye in Fig. 1h. To increase loading volume and improve the application in humans, we designed an accessory device composed of a protective cap, a clear plastic film, a rigid base, and a soft rubber gasket for patient use (Fig. 1i).

### SSOE is biocompatible with cultured human corneal cells and safe to mouse eyes in vivo

**Fig. 2:**
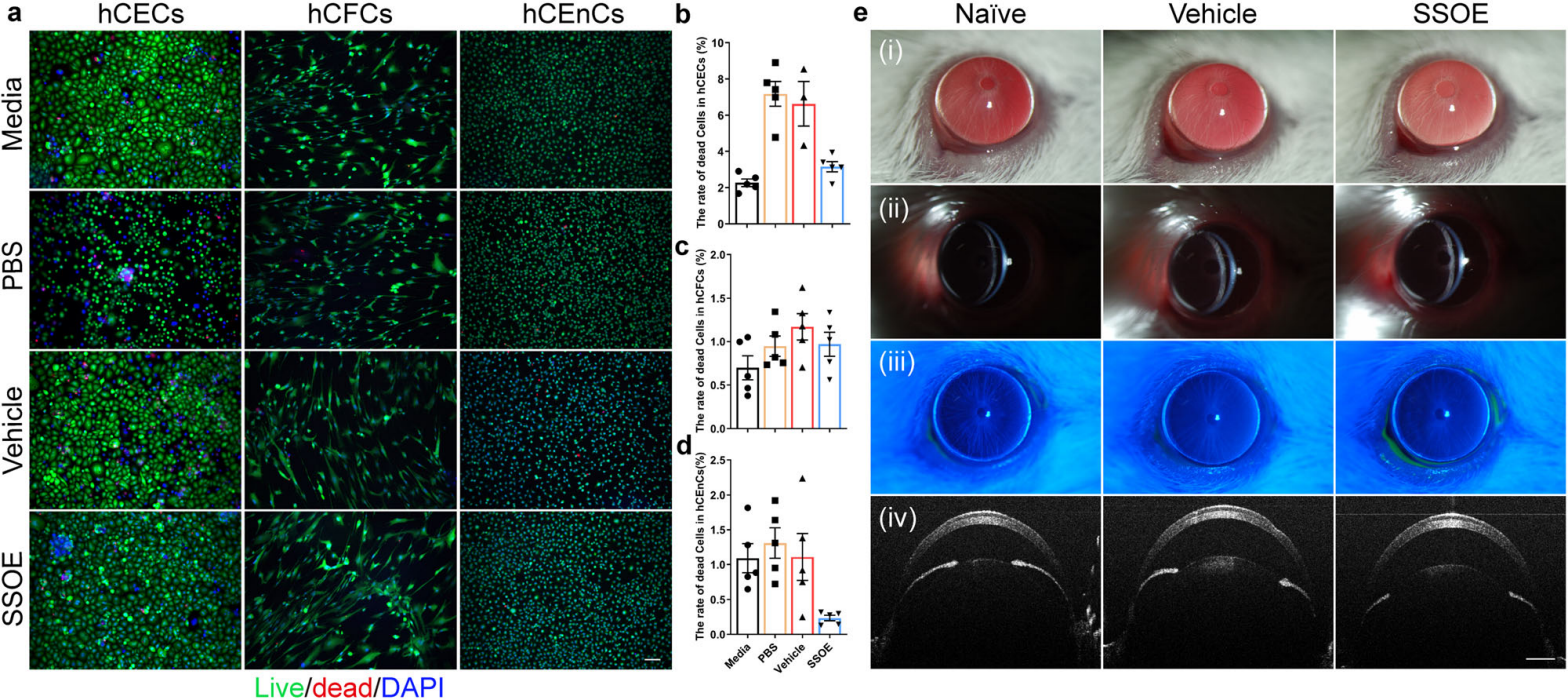
Safety and biocompatibility of ophthalmic SSOE. **a** Representative images of live/dead assay of human corneal epithelial cells (hCECs), human corneal fibroblast cells (hCFCs), and human corneal endothelial cells (hCEnCs) after 1 h exposure to culture media, PBS, unoxygenated vehicle control, or SSOE (scale bar represents 100 μm). Percentages of dead cells in hCECs (**b**), hCFCs (**c**), and hCEnCs (**d**) in SSOE group were comparable or lower than those in culture media and controls. Data are presented as mean ± SEM. hCECs: *n* = 5, 5, 3, 5 individual samples in media, PBS, vehicle and SSOE-treated groups, respectively; hCFCs and hCEnCs: *n* = 5 individual samples in each group. **e** Slit lamp photography showing no signs of ocular redness, irritation, or inflammation 4 h after topical application of SSOE or vehicle controls. (i) Broad beam lighting, (ii) slit beam lighting, (iii) fluorescein staining, and (iv) anterior segment-OCT. Data are representative of three independent experiments. Source data are provided as a Source Data file.

To investigate the safety of SSOE for ophthalmic use, we firstly tested its toxicity in cultured corneal cells. The cornea is the outermost layer of the eye, serving as an important structural barrier and refractive medium, and is composed of the epithelium, stroma, and endothelium. We applied PBS control, unoxygenated emulsion (vehicle) control, or SSOE to cultured human corneal epithelial cells (hCECs), stromal fibroblast cells (hCFCs), and endothelial cells (hCEnCs) for 1 h and determined cell death using Live/Dead Assay. SSOE, but not PBS and vehicle controls, significantly increased the oxygen concentration in cell culture (Supplementary Fig. S1). In all three cell types, cell death rates in SSOE groups were comparable or lower than those of PBS and vehicle controls (Fig. 2a–d). Although SSOE resulted in a statistically significant increase in cell death compared to full culture media in hCECs, the increase is biologically small (3.16 ± 0.28% in SSOE vs 2.27 ± 0.21% in media, Fig. 2b).

We then performed a modified Draize test, a standard test to evaluate the ocular toxicity of medications^19^, in mice. SSOE or vehicle control were applied topically on mouse eyes for 1 h, followed by irrigation, and these mice were observed for one week. Slit lamp photography at 4 h after application (Fig. 2e) did not show eyelid irritation or redness, conjunctival injection or discharge, corneal opacification, or edema. There were no signs of intraocular inflammation, anterior chamber exudation, nor loss of lens transparency. In addition, there was no corneal fluorescein staining, indicative of intact epithelial barrier function (Fig. 2eiii). Optical coherence tomography (OCT) imaging did not show changes in corneal thickness or anterior chamber depth, nor any structural changes in the eye (Fig. 2eiv). Similarly, at 24 h and 7 days post application, no ocular irritation or toxicity were seen (Supplementary Fig. S2).

### SSOE accelerates corneal epithelial wound healing after alkali burn

**Fig. 3:**
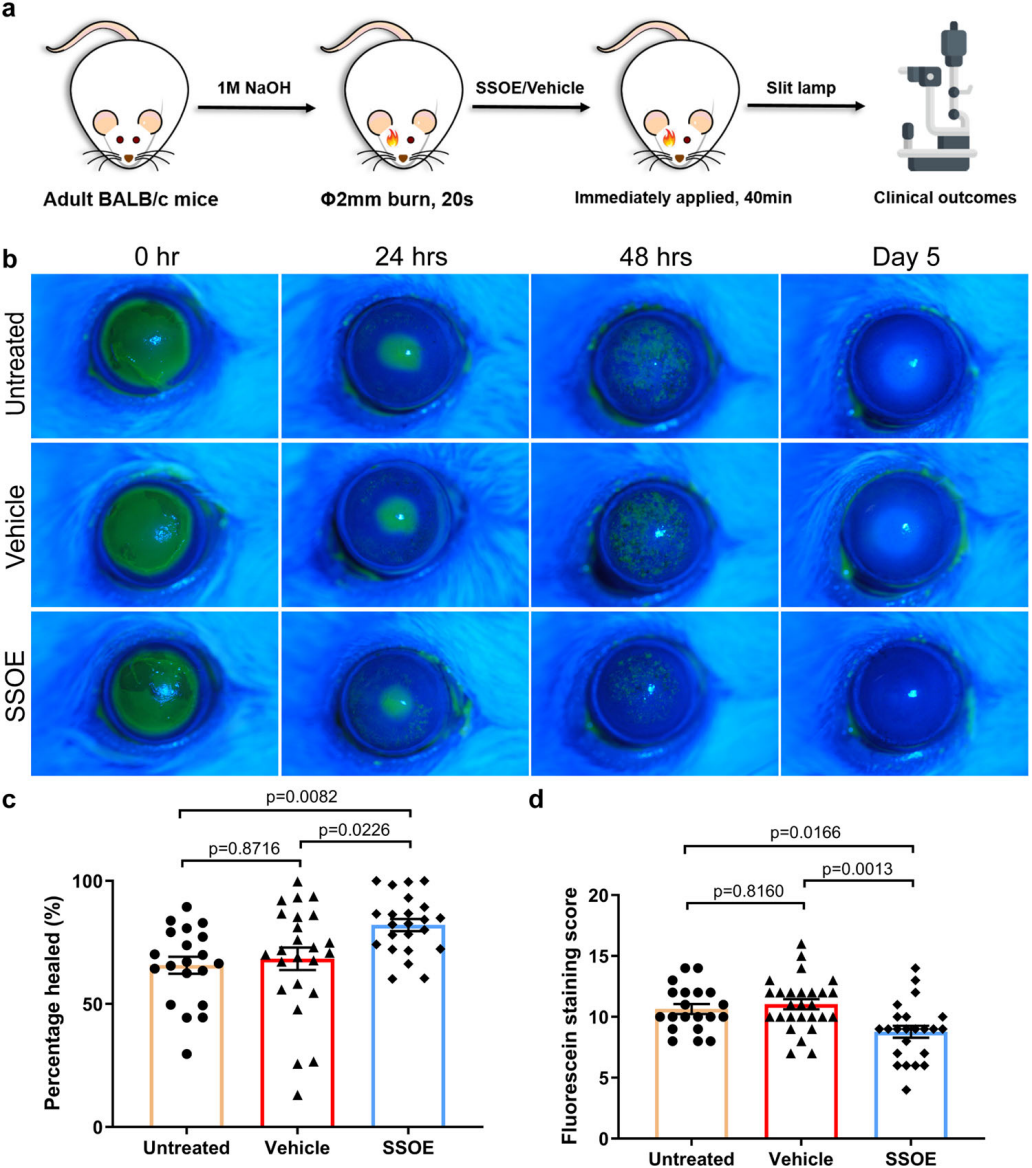
SSOE accelerates corneal epithelial wound healing after alkali burn. **a** Schematic depicting ocular alkali burn in mice: a 2-mm circular filter paper soaked with 1 M sodium hydroxide (NaOH) was applied to the center of the right eye for 20 s, followed by copious irrigation till the pH level returned to normal. SSOE or unoxygenated vehicle control were then immediately applied to the eye for 40 min. **b** Fluorescein staining (green color when photographed with Cobalt blue filter) highlights corneal epithelial defects at 24 h and superficial punctate keratitis at 48 h after burn. SSOE moderately accelerated wound closure at 24 h (**c**) and reduced superficial punctate staining at 48 h (**d**). Data are presented as mean ± SEM. Twenty-four-hour timepoint: *n* = 20, 24, 23 eyes in untreated, vehicle, and SSOE-treated groups, respectively; 48 h: *n* = 20, 27, 23 eyes in untreated, vehicle and SSOE-treated groups, respectively. Statistical significance was determined using one-way ANOVA with Tukey’s multiple comparisons test (two-sided). Data were summarized from seven independent experiments. Source data are provided as a Source Data file.

To evaluate the efficacy of SSOE in treating acute burn, corneal burns were induced using sodium hydroxide (NaOH), followed by copious irrigation in adult BALB/c mice. SSOE or unoxygenated vehicle control (Vehicle) were then immediately applied to the ocular surface for 40 min while mice were under anesthesia. The emulsion (SSOE or vehicle) was then washed off and antibiotic eye ointment was placed in all groups. These mice received no additional treatment afterward. Mice that received ocular burn but not SSOE or vehicle treatment were also included as untreated controls. The ocular changes were evaluated clinically and documented with slit lamp photography (schematic of experiments in Fig. 3a).

In the immediate post-burn period, untreated and vehicle-treated eyes had significant corneal epithelial defects, evidence by fluorescein staining (Fig. 3b). SSOE treatment moderately and significantly accelerated epithelial wound healing at 24 h (Fig. 3c, *P* = 0.0003, compared with the untreated group; *P* = 0.0122, compared with vehicle control group). At 48 h, corneal epithelial defects in all three group were healed but the untreated and the vehicle groups had more punctate staining than the SSOE-treated group (Fig. 3d), indicating better preserved epithelial barrier function (tight junctions) by SSOE treatment. On day 5 after burn, there was no staining in any of the three groups (Fig. 3b).

### SSOE reduces optical opacification and cataract formation after alkali burn

**Fig. 4:**
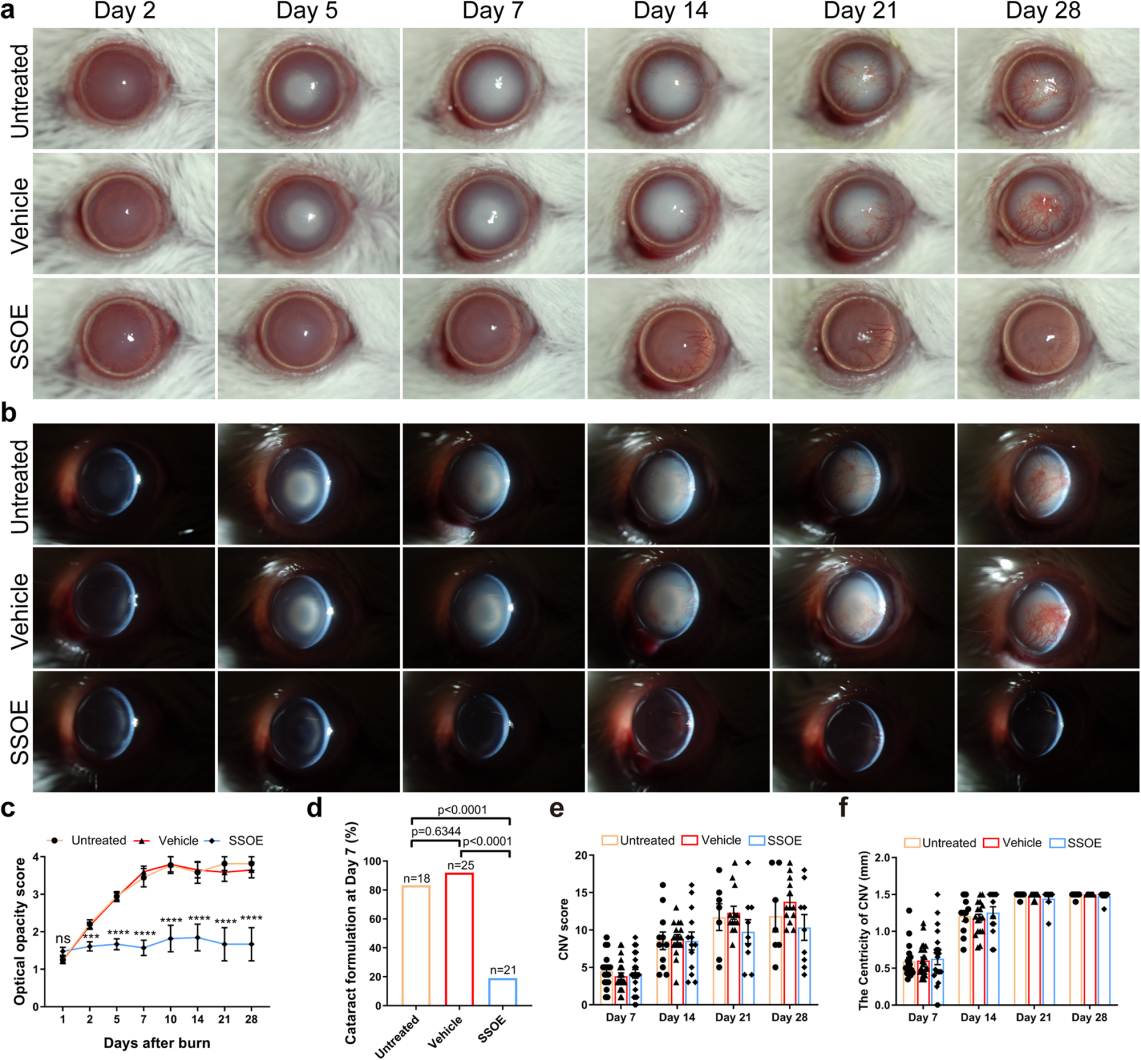
SSOE reduces optical opacification and cataract formation after alkali burn. Representative slit lamp photographs of untreated, vehicle-, and SSOE-treated eyes up to 28 days after burn using broad beam lighting (**a**) and slit beam lighting (**b**). **c** Progressive worsening of optical opacity in untreated and vehicle-treated eyes after burn, while SSOE-treated eyes maintained optical clarity. Data are presented as mean ± SEM. *P* = 0.1537 at day 1 timepoint, ***P = 0.0004, ****P < 0.0001. Statistical significance was determined using one-way ANOVA with Tukey’s multiple comparisons test (two-sided). **d** At day 7 after burn, over 80% of eyes in untreated and vehicle-treated groups developed visually significant cataract, compared to less than 20% in SSOE group. Statistical significance was determined using Fisher’s exact test. **e** Corneal neovascularization (CNV) score and centricity (**f**, length of longest blood vessel from limbus) were comparable among all three groups. Data are presented as mean ± SEM. Days 1 and 2: *n* = 20, 25, 23 eyes in untreated, vehicle and SSOE-treated groups, respectively; days 5 and 7: *n* = 18, 25, 21 eyes in untreated, vehicle and SSOE-treated groups, respectively; day 10: *n* = 9, 10, 11 eyes in untreated, vehicle and SSOE-treated groups, respectively; day 14: *n* = 11, 17, 13 eyes in untreated, vehicle and SSOE-treated groups, respectively; days 21 and 28: *n* = 7, 13, 9 eyes in untreated, vehicle and SSOE-treated groups, respectively. There is no significance in CNV and centricity among untreated, vehicle and SSOE-treated groups (*P* > 0.05). Statistical significance was determined using one-way ANOVA with Tukey’s multiple comparisons test (two-sided). Data were summarized from seven independent experiments. Source data are provided as a Source Data file.

After alkali burn, untreated and vehicle-treated eyes progressively and significantly lost optical clarity of the eye due to a combination of corneal opacification and cataract formation (Fig. 4a, b). SSOE treatment drastically prevented the loss of optical transparency, resulting in significantly lower opacity scores as early as the second day after the burn and this effect lasted up to 1 month (Fig. 4c, *P* < 0.0001). In the untreated and vehicle-treated eyes, serial slit lamp photography demonstrated the gradual clouding of the crystalline lens after burn. By slit lamp examination and photography, the cataract was found to be mostly nuclear sclerosis of the lens. By Day 7, the rates of visually significant cataract in the untreated and vehicle-treated groups were 83.3% and 92.0%, respectively, significantly higher than the rate of 19.0% in SSOE-treated eyes (Fig. 4d, *P* < 0.0001). Of the 21 SSOE-treated eyes, 17 did not develop visually significant cataract throughout the 28-day study duration.

We also examined the formation of new blood vessels (neovascularization) in the normally avascular corneas. Corneal neovascularization began at the limbus as early as 2 days after burn and extended to the center on days 21–28 (Fig. 4e). SSOE treatment resulted in a small and statistically non-significant decrease in neovascularization scores at days 21 and 28 (Fig. 4e). The centricity of these vessels (length of the longest vessel from the limbus) was comparable among all three groups at all timepoints, indicating that SSOE treatment had limited effect on reducing corneal neovascularization (Fig. 4f).

### SSOE reduces ocular inflammation and preserves tissue integrity after alkali burn

**Fig. 5:**
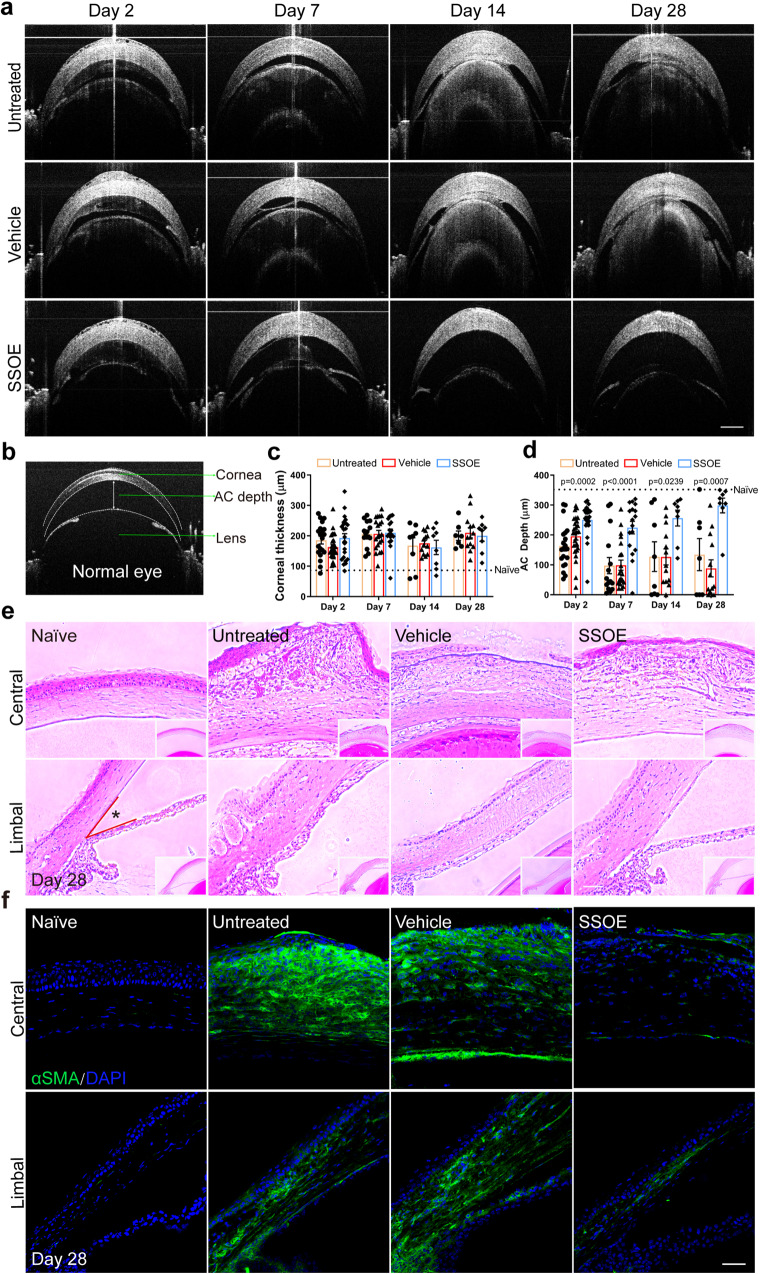
SSOE preserves ocular anatomical and tissue integrity after alkali burn. **a** Representative OCT images depicting reduced anterior chamber (AC) exudation and adhesion of the iris/lens to the cornea in SSOE group. **b** OCT image of a normal mouse eye. AC depth is measured centrally between the posterior surface of the cornea and the iris plane. **c** Persistent corneal edema was observed and comparable in all three groups (dashed line representing normal corneal thickness). **d** AC depth was maintained in SSOE-treated eyes, while decreased in untreated and vehicle-treated eyes (dashed line representing normal AC depth). Data are presented as mean ± SEM. Statistical significance was determined using one-way ANOVA. There is no significance in corneal thickness among untreated, vehicle and SSOE-treated groups (*P* > 0.05). Day 2: *n* = 20, 27, 23 eyes in untreated, vehicle and SSOE-treated groups, respectively; day 7: *n* = 15, 21, 17 eyes in untreated, vehicle and SSOE-treated groups, respectively; day 14 and 28: *n* = 8, 13, 8 eyes in untreated, vehicle and SSOE-treated groups, respectively. **e** H&E staining showing central (top panel) and limbal (bottom panel) structures of the cornea. SSOE treatment led to reduced leukocyte infiltration, tissue fibrosis, and loss of AC angle between the cornea and iris root (*) (scale bar represents 50 μm). Data are representative of three independent experiments. **f** Alpha-smooth actin (αSMA, green) immunostaining highlights reduced tissue fibrosis in the central (top panel) and limbal (bottom panel) areas of the cornea in SSOE group (scale bar represents 50 μm). Data are representative of three independent experiments. Source data are provided as a Source Data file.

To investigate the effect of SSOE on ocular micro-anatomy and tissue integrity after alkali burn, serial OCT imaging was performed in live mice (Fig. 5a). Figure 5b shows the normal anatomy of the anterior segment of the eye, including the cornea, iris, lens, and the anterior chamber (formed between the cornea and iris/lens). Alkali burn resulted in persistent corneal edema, evidenced by increased corneal thickness (Fig. 5a) and neither vehicle nor SSOE treatment had significant effects on corneal edema at any timepoint (Fig. 5c). Alkali burn led to significant exudation within the anterior chamber, followed by a subsequent narrowing of the chamber, and in severe cases adhesion of the iris/lens to the cornea (anterior synechiae) (Fig. 5a). SSOE, but not vehicle treatment, resulted in much reduced exudation and maintained the anterior chamber depth (~300 μm) close to that of normal mice (350 μm) (Fig. 5d).

The eye was also subjected to histopathological analysis. H&E staining (Fig. 5e) showed massive leukocyte infiltration, tissue fibrosis, and distortion of the cornea, as well as significant exudation within the anterior chamber and adhesion of the iris/lens to the cornea in the untreated and vehicle-treated eyes on day 28 after burn. In SSOE-treated eyes, alkali-induced infiltration of inflammatory cells into the cornea was reduced, the corneal stroma was less disorganized, and the anterior chamber was maintained (Fig. 5e, top panel). The angle of the anterior chamber (*Fig. 5e, lower panel), where the corneal endothelium and the root of iris meet, is obliterated in untreated and vehicle-treated eyes; whereas it was maintained in the SSOE-treated ones.

α-smooth muscle actin (αSMA), a marker for myofibroblasts, highlights tissue fibrosis and scar formation. The anterior segment of the eye, including the cornea, is void of αSMA staining in normal condition. In untreated and vehicle-treated eyes after alkali burn, there was intense αSMA staining throughout the entire cornea and in part of the iris (Fig. 5f). SSOE treatment on the other hand resulted in scarce αSMA staining.

### SSOE reduces ocular tissue hypoxia and cell death after alkali burn

**Fig. 6:**
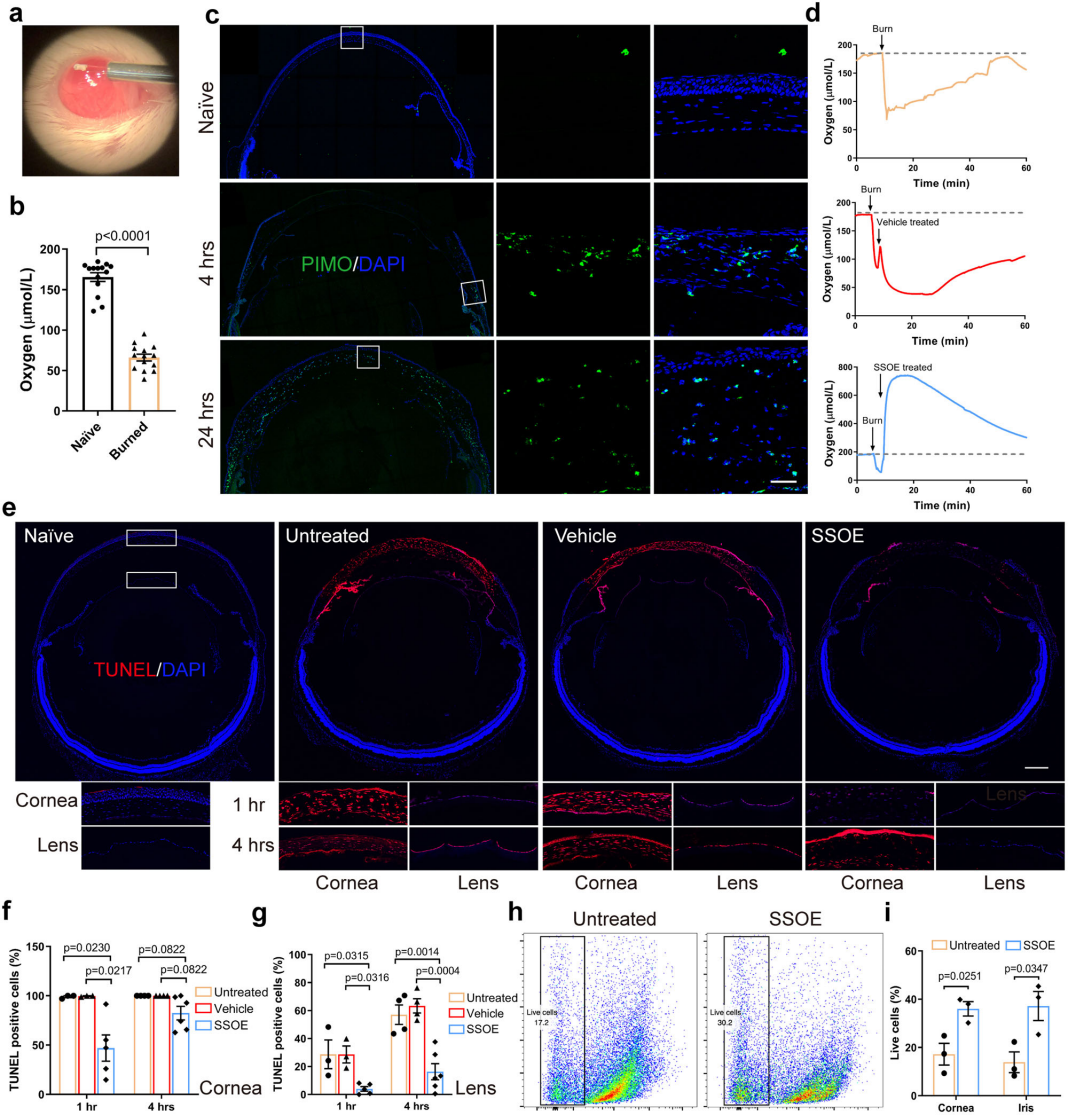
SSOE reduces ocular tissue hypoxia and cell death after alkali burn. **a** Oxygen concentration in anterior chamber (AC) was recorded in real time by inserting a micro-oxygen sensor. **b** AC oxygen level decreased by 60% immediately after alkali burn. Data are presented as mean ± SEM. Statistical significance was determined using unpaired, two-tailed Student’s *t* test. *n* = 14 eyes in each group. **c** Pimonidazole hydrochloride (PIMO) staining, a chemical hypoxia marker, showed limbal staining at 4 h post burn and diffuse staining in the entire cornea by 24 h (inserts showed higher magnification of selected areas in white boxes, scale bar 10 μm). Data are representative of three independent experiments. **d** In untreated and vehicle-treated eyes, AC oxygen concentration decreased rapidly after burn and gradually increased within one hour. SSOE raised AC oxygen concentration to supra-physiologic level after burn and maintained high levels for more than 60 min. Dashed line represents baseline AC oxygen concentration. **e** Whole mouse globe was cryo-sectioned and TUNEL (red) staining highlighted dead cells in the eye at 1 h after alkali burn in untreated and vehicle groups. SSOE treatment reduces the extent of cell death. Lower panels show higher magnification of the central cornea and anterior lens capsule at 1- and 4 h post burn (scale bar 0.3 mm). The percentage of TUNEL-positive cells in the cornea (**f**) and lens epithelium (**g**) was lower in SSOE group. Data are presented as mean ± SEM. 1 h timepoint: *n* = 3, 3, 5 eyes in untreated, vehicle and SSOE-treated groups, respectively; 4 h: *n* = 4, 4, 6 eyes in untreated, vehicle and SSOE-treated groups, respectively. Statistical significance was determined using one-way ANOVA with Tukey’s multiple comparisons test (two-sided). **h** The percentage of live cells in the cornea 1 h post burn was determined by flow cytometry. **i** There were significantly more live cells in the cornea and iris of SSOE-treated eyes compared to untreated eyes. Data are presented as mean ± SEM. Statistical significance was determined using unpaired, two-tailed Student’s *t* test. *n* = 3 eyes in each group. Source data are provided as a Source Data file.

Consistent with the previous reports^6^, using a micro-oxygen sensor (Fig. 6a), we demonstrated a 60% decrease in oxygen concentration within the anterior chamber seconds after alkali burn (165.4 ± 20.3 μmol/L to 66.1 ± 15.6 μmol/L, *P* < 0.0001, Fig. 6b). Using pimonidazole hydrochloride, a hypoxia marker, we observed cellular hypoxia (indicated by green staining) at the limbal area as early as 4 h post burn (Fig. 6c), and this propagated to the entire cornea by 24 h. We observed no pimonidazole staining in the retina. Recording in real time, we observed a sharp decline in oxygen level after burn and the level gradually returned to the normal range in 50 min (Fig. 6d). While in vehicle-treated eyes, anterior chamber oxygen concentration remained below baseline level up to 60 min, SSOE treatment led to a rapid and robust increase in oxygen concentration with peak level of 534.8 ± 164.7 μmol/L in a few seconds, and such increase maintained above baseline level for more than 60 min.

Hypoxia leads to cell death^20,21^. Next, we examined cell death using TUNEL staining after alkali injury (Fig. 6e–g). At 1 h after burn, nearly all cells in the central corneas of untreated or vehicle-treated eyes were TUNEL-positive. In SSOE-treated eyes, the TUNEL-positive cells were seen mostly at the superficial epithelial layer, which was in direct contact with NaOH. Less TUNEL staining was observed in the iris of the SSOE-treated eyes, compared with untreated and vehicle-treated eyes. As the impact of alkali injury propagated beyond the cornea at 4 h, the lens epithelial cells lining up the anterior lens capsule stained positive for TUNEL in untreated and vehicle-treated eyes; whereas there was little TUNEL positivity in the lens of SSOE-treated eyes. We did not observe TUNEL-positive cells in the retina or optic nerve at these timepoints (Fig. 6e). To confirm the results of TUNEL staining, we performed flow cytometry (Fig. 6h, i) and found the percentage of live cells in the cornea was significantly higher in SSOE-treated group (36.0 ± 3.0%), compared to the untreated eyes (17.2 ± 4.5%) 1 h post burn. Similar changes were observed in the iris as well (37.2 ± 6.0% in SSOE vs 13.9 ± 4.3% in the untreated group).

### SSOE reduces leukocyte infiltration, production of inflammatory mediators, and hypoxia-inducible factor 1-alpha signaling after alkali burn

**Fig. 7:**
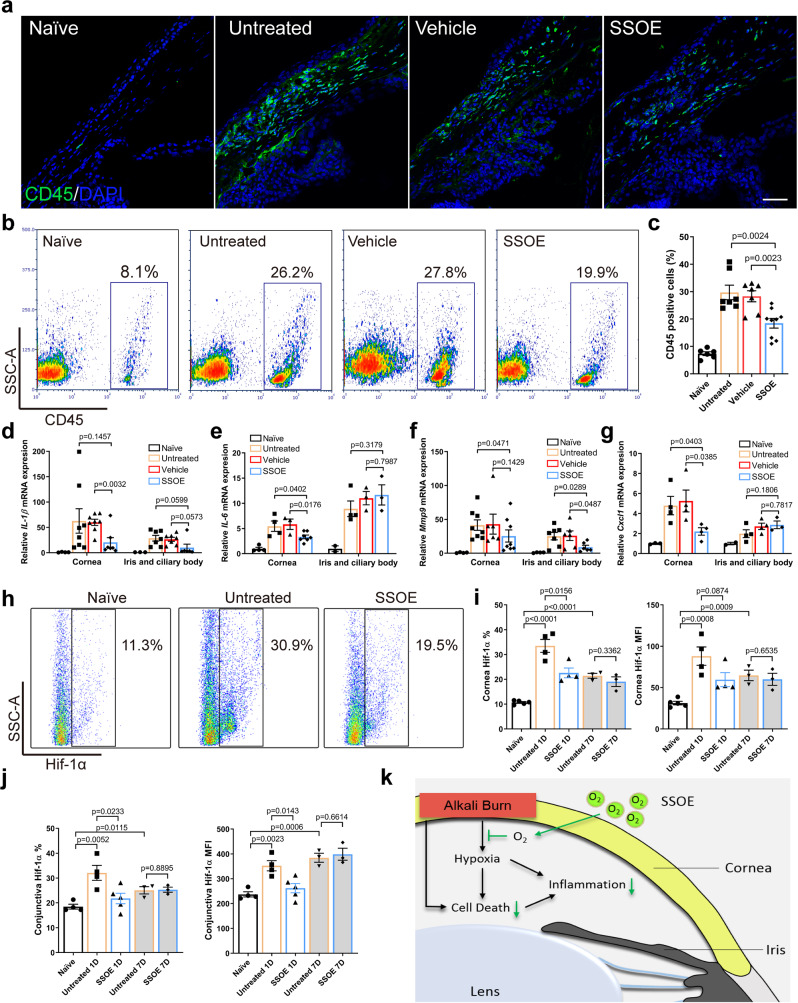
SSOE reduces ocular tissue inflammation and hypoxia-inducible factor 1-alpha (Hif-1α) signaling after alkali burn. **a** Alkali burn led to massive infiltration of CD45^+^cells in limbal area at 24 h post burn and SSOE reduced the number of infiltrating cells (scale bar 50 μm). **b** SSOE treatment led to a decrease in CD45^+^ cells in the conjunctiva, assessed with flow cytometry. **c** Quantification of CD45^+^ percentage. *n* = 8, 9, 9, 11 eyes in naive, untreated, vehicle and SSOE groups. *IL-1β* (**d**), *IL-6* (**e**), *Mmp9* (**f**), and *Cxcl1* (**g**) mRNA levels in the cornea were increased 24 h post burn and SSOE treatment decreased their levels. While the levels of these cytokines were also increased in the iris/ciliary body after the burn, only MMP9 levels were significantly decreased by SSOE treatment. *IL-1β*: *n* = 4, 8, 8, 7 corneas/3, 7, 7, 6 iris and ciliary bodies in naive, untreated, vehicle and SSOE; *IL-6*: *n* = 4, 4, 3, 7 corneas/2, 4, 3, 3 iris and ciliary bodies; *Mmp9*: *n* = 4, 8, 6, 7 corneas/4, 7, 6, 6 iris and ciliary bodies; *Cxcl1*: *n* = 4, 4, 4, 4 corneas/2, 4, 4, 3 iris and ciliary bodies. **h** Hif-1α level in the cornea at 1 day post burn increased significantly. **i** SSOE treatment reduced the percentage (%) and mean fluorescent intensity (MFI) of Hif-1α in the cornea at 1 day (1D), but not 7 days (7D) post burn. *n* = 5, 4, 4, 3, 3 eyes in naive, untreated 1D, SSOE 1D, untreated 7D and SSOE 7D groups, respectively. **j** SSOE treatment reduced % and MFI of Hif-1α in the conjunctiva at 1D but not 7D post burn. Data are presented as mean ± SEM. *n* = 4, 4, 5, 3, 3 eyes in naive, untreated 1D, SSOE 1D, untreated 7D and SSOE 7D groups, respectively. **k** Schematic showing SSOE mitigating ocular alkali burn by reducing tissue hypoxia, cell death, and inflammation. Data are presented as mean ± SEM. Statistical significance was determined using unpaired, two-tailed Student’s *t* test. Source data are provided as a Source Data file.

Lastly, we examined the cellular and molecular mechanisms underlying the efficacy of SSOE treatment. Alkali burn induced significant CD45^+^ leukocyte infiltration from the conjunctiva to the peripheral cornea at 24 h and SSOE treatment led to a reduction. (Fig. 7a). Consistent with immunohistochemical staining, we observed much higher percentage of CD45^+^ cells in the conjunctiva in the untreated (29.7 ± 7.0%) and vehicle-treated (28.3 ± 5.3%) eyes, compared to SSOE-treated ones (18.4 ± 5.2%) using flow cytometry (Fig. 7b, c). In addition, we detected increases in the expression of inflammatory cytokines IL-1β and IL-6, inflammatory mediator MMP9, and chemokine Cxcl1 (Fig. 7d–g, respectively) in the cornea, as well as iris/ciliary body after burn. SSOE, but not vehicle treatment, decreased their levels in the cornea significantly. SSOE also reduced levels of IL-1β and MMP9 in the iris and ciliary body.

Hypoxia-inducible factor (Hif) transcription factors are master regulators of the tissue and cellular response to hypoxia. Since SSOE treatment reversed tissue hypoxia induced by alkali burn, we sought to determine Hif-1-alpha (Hif-1α) signaling using flow cytometry (Fig. 7h–j). In response to alkali burn, the percentage of Hif-1α positive cells (33.5 ± 2.6% in untreated vs 10.7% ± 0.3% in naive) in the cornea and their protein levels (MFI, 88.1 ± 11.2 in untreated vs 31.2 ± 2.2 in naive) increased significantly at 1 day post burn (Fig. 7i). SSOE treatment led to a decrease in these levels (22.6 ± 2.0% and 59.6 ± 8.4 for MFI). By 7 days post burn, Hif-1α signaling in the cornea remained elevated (21.4 ± 1.0% and 64.7 ± 6.4 for MFI), albeit to a lesser extent compared to 1 day, and there were no significant differences between the untreated and SSOE-treated eyes. Similar changes in Hif-1α signaling in the conjunctiva were observed (Fig. 7j). In addition, we investigated levels of growth factors expressed in the cornea, conjunctiva, and iris at 7 days post burn (Supplementary Fig. S4) and found no significant changes in an array of 30 growth factors, except for a decrease in the level of insulin-like growth factor type 1 receptor by SSOE treatment.

## Discussion

Herein, we took advantage of the beneficial effect of oxygen in burn treatment by developing a perfluorodecalin-based oxygen emulsion for topical ophthalmic use. The emulsion releases oxygen rapidly and sustainably. It maintains oxygen-releasing capacity in room-temperature storage up to 1 year. It is packaged in a pressurized canister, which can be aimed and dispensed directly into the injury site without the need for additional tools. It remains on/in the injured tissues and continues to release oxygen over hours, reducing the need for repeated application. It is compatible with ocular surface cells and shows no ocular toxicity in live animals. Most importantly, a single topical application of SSOE significantly reduces tissue hypoxia, inflammation, cell death, and Hif-1α signaling, leading to less cataractogenesis and ocular opacity, and faster corneal epithelial wound healing after alkali burn (schematic of SSOE in treating alkali burn in Fig. 7k). In conclusion, our findings provide proof-of-concept data that a perfluorodecalin-based supersaturated oxygen emulsion (SSOE) is a safe, effective, and ready-to-use topical therapeutic in treating acute alkali burn to the eye.

Oxygen, a naturally occurring atmospheric gas indispensable to most living organisms, is a key substrate in cellular metabolism and bioenergetics. Systemic oxygen therapy has been shown to reduce disease severity after chemical injury in animals and humans^7,8^. To overcome the limitation of systemic oxygen delivery, attempts to harvest and store oxygen bound to a carrier have been made. Perfluorocarbons (PFCs) are inert hydrophobic chemicals that dissolve gas, including oxygen, carbon dioxide, and nitrogen^22^. PFCs do not “bind” gases but rather dissolve them with high capacity. This is due to the weakness of the cohesive forces between PFC molecules, which facilitate the formation of ‘hole’s that accommodate gas molecules. At a given temperature, the solubility of a given gas in a PFC is directly proportional to it partial pressure^22^. We originally developed this perfluorodecalin (PFD, 35 and 55%)-based oxygen emulsion as a topical skin wound healing technology^16^. In the current study, we formulated the emulsion for ophthalmic application by reducing PFD concentration to 25% and removing preservatives, leaving only two surfactants and water, in addition to PFD, in SSOE. For the topically applied SSOE to increase local oxygen concentration, there are two important mechanisms of chemical species dissolution via PFC phases and transport at the colloidal suspension tissue interface at play. First, the PFD molecules inside colloidal moieties dissolve oxygen much more readily than the non-PFD aqueous phase, allowing for much higher oxygen concentrations. A difference in concentration will exist between the aqueous phase and the PFD-rich particles. Second, there is a gradient and a movement of oxygen from the colloidal particles (in this case pressurized in a vessel) to living cells/tissues and the atmosphere. It is worth noting that PFCs including PFD dissolve and change the concentrations of other gases such as CO_2_ and nitrogen, as well as other biologically relevant oxygen-containing species^12,22–24^. Therefore it is perceivable that some of the observed biological results of SSOE treatment may be related to the PFD. To overcome this confounding issue, we have used unoxygenated PFD emulsion as the vehicle control throughout the study and the vehicle control group demonstrated similar results to the untreated controls. Therefore, the treatment effects of SSOE observed in the current study are mostly due to the oxygen delivered, rather than the PFD.

PFD liquid has been used in ophthalmic surgery to mechanically tamponade detached retina due to its high specific gravity and it is removed at the end of the surgery^15,25^. There are contradictory reports regarding its toxicity to the retina: while some reported retinal ganglion and retinal pigmented epithelial cell death with prolonged contact with PFD liquid beyond 2 days^26,27^, others reported no structural damages after 3-month vitreous tamponade in rabbits^28^. Several cases of corneal toxicity due to direct contact between PFD droplets and cornea endothelium have been reported^29^. It is worth noting that these agents used in vitreoretinal surgery are pure high-gravity liquids with PFD concentration above 80% (often 100%) and the toxicities are almost all due to the PFD liquid inadvertently or experimentally left in the eye for a prolonged time. The SSOE formulation used in our study contains 25% PFD and its application is for the ocular surface and transient (less than 1 h contact time with the eye in each application). Our data demonstrate the lack of toxicity of SSOE to cultured human corneal cells and more importantly to the intact mouse eyes in vivo (observed for 1 week after a single application).

Consistent with the previous report^6^, we found rapid and significant reduction (~60%) in the oxygen concentration in the aqueous humor after alkali burn. Hypoxia directly leads to tissue damage, and more importantly triggers leukocyte infiltration and the secretion of inflammatory cytokines^30^. The infiltrated leukocytes in turn exacerbate tissue hypoxia, compounding tissue damage. Within seconds of application, SSOE reverses hypoxia in the aqueous humor after alkali burn, raising oxygen concentration to supra-physiologic levels for more than one hour. Within one hour of alkali burn, there is significant cell death throughout the entire cornea (the epithelium, stroma, and endothelium), rapidly propagating to the iris and anterior lens capsule within 4 h. SSOE treatment significantly limits cell death in the cornea, iris, and anterior lens capsule. It is conceivable that the early cell death in the lens epithelial cells is the initial signal for cataract formation after alkali burn, and cataract progression is sustained by the ongoing inflammation. In addition, SSOE reduces early and delayed leukocyte infiltration into the burned cornea and the rest of the anterior segment of the eye (conjunctiva, iris and ciliary body). Proinflammatory cytokine IL-1β, IL-6, chemokine Cxcl1, and inflammatory mediator matrix metalloproteinase-9 (MMP-9, a key enzyme for corneal extracellular matrix degradation) are also reduced with SSOE treatment. These data clearly demonstrate that SSOE reveres acute tissue hypoxia, early cell death, and inflammation after alkali burn. Interestingly, we found no significant changes in the expression of a panel of 30 growth factors in the cornea, conjunctiva, and iris with SSOE treatment 7 days after burn, except for a reduction in insulin-like growth factor type 1 receptor level. This suggests that the effect of a single application of SSOE is likely acute and has limited impact on tissue regeneration in the long run.

We observed robust therapeutic efficacy of SSOE in reducing disease burden after acute burn: accelerated epithelial wound healing, diminished anterior chamber exudation and fibrosis, and reduced optical opacification and cataract formation. Posterior segment damage, including the development of glaucoma and loss of retinal ganglion cells after alkaline burn has been reported^6^. In our study, we did not observe structural alterations on H&E staining or cell death by TUNEL staining in the retina or optic nerve. The discrepancy may be due to the difference in burn severity.

Hypoxia triggers a multitude of cellular and molecular changes to adapt to the lack of oxygen, particularly activating the hypoxia-inducible factor (Hif) signaling pathways^31^. While this pathway has been well studied in multiple systems, its role in the cornea is less clear^32^. In the current study, we found that alkali burn induces Hif-1α activation at 1- and 7-day post burn in the cornea and conjunctiva, and that SSOE treatment effectively inhibits this activation at 1 day but not 7-day. Since SSOE treatment in the current study was a one-time application for less than 60 min immediately after burn, it is perceivable that its impact on Hif-1α signaling is transient. Interestingly, despite an initial reversal of acute tissue hypoxia and reduction in Hif-1-α signaling, we found no changes in corneal neovascularization in the 1-month follow-up period. Others have reported that silencing Hif-1-α reduced corneal neovascularization secondary to alkali burn^33^ and herpetic stromal keratitis^34^, possibly via modulating VEGF levels^33,35^, although Hif-1-α-independent VEGF activation has been noted in the corneal stromal cells^36^. In our study, the lack of effect on corneal neovascularization by SSOE can be explained by some of the following reasons. First, inhibition of Hif-1α signaling by SSOE is transient (1 day, but 7 days post burn), therefore not long enough to suppress later rises in Hif signaling and subsequent VEGF activation. Second, this is actually consistent with previous reports that external oxygen up to 75% concentration at the ocular surface does not affect corneal neovascularization after cautery injury^37^. Third, there may be a hypoxia- or Hif-1α-independent VEGF activation pathway and angiogenic stimulus in the current model.

Rao et al. recently reported that inhibition of Hif signaling had divergent effects on corneal neovascularization and opacity in that acriflavine inhibiting both Hif-1α and 2α decreased neovascularization but increased opacity in HSV-1-infected corneas^34^. Our study observed much reduced ocular inflammation and opacification (in the cornea and lens) and no effect on corneal neovascularization by SSOE treatment. As Hif downstream signaling encompasses a wide variety of vascular, inflammatory, and fibrotic responses, these studies suggest that Hif inhibition may have differential effects on individual pathological process. In addition, Rao et al. showed that Hif-1α and 2α were detected in infiltrating immune cells and corneal epithelial cells, respectively, in herpes stromal keratitis; and their inhibition by acriflavine led to a decreased influx of CD4 T cells and nongranulocytic myeloid cells, but an increased influx in neutrophils, into the infected area^34^. Our study demonstrated reduced overall CD45^+^ infiltration into the burned cornea with SSOE treatment but did not elucidate the changes in the specific population of these infiltrate leukocytes. Consistent with Rao’s report, we also detected Hif-2α signaling in the corneal epithelium, but its levels were not changed with alkali burn or SSOE treatment. Therefore, the role of Hif-2α signaling in alkali burn and the effect of SSOE on this pathway are less clear.

In addition to Hif, SSOE may impact other signaling pathways, particularly reactive oxygen species (ROS) and nitric oxide (NO) production. In our exploration into these areas, we found that alkali burn led to a rapid increase in H_2_O_2_ levels in the aqueous humor and SSOE was able to significantly reduce its levels. We were unable to detect significant NO levels in the aqueous humor. ROS generation offers an additional potential mechanism by which SSOE treatment alleviates inflammation, fibrosis, and cataractogenesis. ROS and Hif signaling are intertwined complex processes. On the one hand, ROS has been shown to regular Hif signaling in hypoxic conditions^38^. On the other hand, Hif signaling is known to play a role in oxidative stress and a reduction in Hif-1α and 2α signaling has been shown to enhance ROS generation^39,40^. In Rao’s study in herpes stromal keratitis^34^, the authors speculated that inhibition of Hif signaling may increase the level of ROS and thereby exacerbates corneal opacity. It is perceivable that SSOE treatment has differential effect on Hif signaling and ROS production via different mediators with different time courses. The exact mechanism warrants further study.

Our study has several limitations. We only tested a single application of SSOE immediately after sodium hydroxide alkali burn. It is conceivable that SSOE has a therapeutic window during which a delayed application is still effective and that SSOE may be effective against other chemical injuries. The current burn is limited to the central 2 mm diameter of the mouse cornea sparing the limbus, therefore the effect of SSOE on limbal stem cell deficiency cannot be evaluated. In addition, compared to larger mammals and humans, rodent eyes are marked by relatively large size of the lens, short distance between the ocular surface and internal ocular tissues, and large size of the ocular surface compared to the volume of the globe^41^, although it has been shown that alkali burn caused similar extents (depths) of intraocular tissue hypoxia and inflammation between mice and rabbits^6^. Although we provided mechanistic insight into the therapeutic efficacy of SSOE, that is the reversal of tissue hypoxia and transient inhibition of Hif signaling, additional examinations into ROS production and various downstream mediators of Hif signaling are not included. For the aforementioned limitations, further studies are warranted, and we are currently exploring delayed application of SSOE in treating alkali ocular burn in rabbits. Lastly, PFD has been used as an oxygen carrier in blood substitutes, some of which have been FDA approved for clinical use but subsequently recalled and/or withdrawn, partially due to their lack of stability over time^22^. Our current study briefly tested the oxygen-releasing capacity of SSOE and showed that it continued to release high concentrations of oxygen when stored at room temperature over the course of one year. Additional rigorous testing of its stability over longer period of time and under different storage and transportation conditions is warranted for its eventual clinical use.

In summary, we developed a topical perfluorodecalin-based supersaturated oxygen emulsion to treat chemical injuries to the eye. SSOE is biocompatible with human corneal cells and safe for ophthalmic use in vivo. It is effective in reducing alkali burn-induced tissue hypoxia, cell death, and excessive intraocular inflammation, leading to preserved ocular integrity and optical transparency. SSOE represents a unique therapeutic for the unmet medical needs in the acute treatment of ocular chemical injuries.

## Methods

### Animals

BALB/c mice were obtained from Charles River Laboratories (Wilmington, MA, USA) and housed in the animal vivarium of the Schepens Eye Research Institute. All animal procedures were performed in accordance with the Association for Research in Vision and Ophthalmology (ARVO) statement for the use of Animals in Ophthalmic and Vision Research and approved by the Animal Care Committee of the Schepens Eye Research Institute. A preliminary study used equal numbers of male and female adult mice and showed no sex-based differences in clinical efficacy. In the current report, adult male mice between the ages of 8 and 10 weeks were used. The mice were kept at a 10/14-h dark/light cycle with ambient temperature 21–23 °C, and humidity 40–60%.

### SSOE ingredients

Perfluorodecalin was distributed by Mel-Co® (Coachella Valley, CA, USA). Phospholipon 90H was from the American Lecithin Company (Oxford, CT, USA). Polawax was from Croda Inc (Edison, NJ, USA).

### Transmission electron microscopy

SSOE emulsion was applied onto carbon and formvar-coated 200 mesh copper grids and air-dried. Grids were imaged using an FEI Tecnai G2 Spirit transmission electron microscope (FEI, Hillsboro, OR, USA) at 100 kV interfaced with an AMT XR41 digital CCD camera (Advanced Microscopy Techniques, Woburn, MA, USA).

### Oxygen measurement

Partial oxygen pressure (pO_2_) in SSOE and cell culture media was measured with DP-PSt7-10 oxygen sensor (PreSens, Regensburg, Germany). In brief, 1 ml SSOE was dispensed into a 3.8-cm^2^ culture plate. The sensor was immediately immersed within the emulsion or culture media and the pO_2_ values were recorded in real time. The anterior chamber oxygen measurement was performed using the DP-PSt7-2 oxygen sensor. Briefly, anesthetized mice were mounted in a stereotaxic frame and the oxygen sensor was immobilized on a manual micromanipulator. A 28-gauge needle was used to perform a narrow, self-sealing tunnel in the temporal region of the cornea, adjacent to the limbus. Before the insertion of the needle tip, it was marked with surgical dye (Accu-line Products Inc., Hyannis, MA, USA) to guide the insertion of the sensor probe into the anterior chamber.

### Ocular alkali burn

Mice were anesthetized using 60 mg/kg ketamine and 6 mg/kg xylazine, and deep anesthesia was confirmed by a toe pinch test. One drop of 0.5% Proparacaine hydrochloride USP (Bausch & Lomb, Tampa, FL, USA) was applied to the right eye for 1 min. A sterile 2-mm-diameter filter paper disc (1001-329, Whatman, Maidstone, UK) was soaked in 1 mol/L sodium hydroxide solution for 10 s. Excess sodium hydroxide was dried by touching the edge of the filter paper disc on a paper towel once. The filter paper disc was then applied onto the central cornea (sparing limbus) for 20 s. After removing the filter paper, the ocular surface was irrigated with PBS via an 18-gauge needle attached to an intravenous infusion bag until the pH level of the ocular surface returned to 7. Mice were then placed on a heating pad, and SSOE or vehicle control emulsion (approximately 50 μl) was applied on the ocular surface of the burned eye for 40 min while the mice were still under anesthesia. After the mice woke up, 0.05 mg/kg Buprenorphine hydrochloride (Reckitt Benckiser Healthcare Ltd, Hull, UK) was administered by subcutaneous injection for pain management and triple antibiotic eye ointment (Bausch & Lomb, Tampa, FL, USA) was applied, topically to prevent infection. No additional ocular treatment was given afterward.

### Clinical evaluation

Corneal fluorescein staining was used to evaluate corneal epithelial damage. Briefly, 1 µl of 2% fluorescein (Sigma-Aldrich, St. Louis, MO, USA) was applied into the lateral conjunctival sac of the mice for 10 s, and then the eye was washed with PBS to remove the excessive fluorescence. The corneas were immediately examined with a slit lamp biomicroscope (Topcon, Tokyo, Japan) under cobalt blue light. Corneal epithelial defects were determined by measuring the stained areas. Corneal epithelial punctate staining was scored using the standard National Eye Institute grading system of 0 to 3 for each of the five areas of the cornea (central, superior, inferior, nasal and temporal) and then totaled (score range 0–15). Optical opacity was scored in 0 to 4, based on the standard photographs in Supplementary Fig. S3. Cornea neovascularization was assessed using a modified scoring system. A score of 0–4 was assigned to each of the five areas of the cornea (central, superior, inferior, nasal and temporal), based on the area occupied by new blood vessels: 0, no new blood vessels; 1, less than 30% area; 2, more than 30% area but less than 70%; 3, more than 70% area less than 100%; and 4, 100% area (range 0–20). The type of cataract was determined by slit lamp biomicroscopy. Intraocular pressure was measured using a tonometer (Icare TONOLAB, Vantaa, Finland). Anterior segment images were taken using anterior segment-optical coherence tomography (OCT) Bioptigen Spectral Domain Ophthalmic Imaging System Envisu R2200 with 12-mm telecentric lens (Bioptigen Inc, Durham, NC, USA). Corneal thickness and anterior chamber depth were measured using the OCT built-in software. Retinal images were taken with the Micron III image-guide system (Phoenix Technology Group, Pleasanton, CA) and retinal thickness was measured using OCT.

### Detection of tissue hypoxia using Hypoxyprobe™

Pimonidazole hydrochloride (HP2-200kit; Hypoxyprobe, Inc., Burlington, MA, USA) was dissolved in 0.9% normal saline and injected intraperitoneally at the final concentration of 1.2 mg/20 g mouse body weight at 4- and 24 h post burn. Mice were euthanized 40 min post injection, and mouse eyeballs were enucleated and frozen embedded in the OCT compound before cryostat sectioning.

### Immunofluorescent staining

Mouse whole eyes were sectioned (9 μm in thickness) and fixed in 4% paraformaldehyde for 20 min. The slides were incubated in 0.2% Triton X-100 for 20 min and then 2% BSA for 1 h at room temperature. Slides were incubated with anti-aSMA (1:200 dilution, 14-6496-82, eBioscience, San Diego, CA, USA) or anti-CD45 (1:100 dilution, 103101, BioLegend, San Diego, CA, USA) antibodies at 4 °C overnight, followed by Alexa Fluor 488-conjugated donkey anti-mouse secondary antibody (1:500 dilution, A-21202, Invitrogen, Carlsbad, CA, USA) or donkey anti-rat secondary antibody (1:500 dilution, A-21208, Invitrogen) for 1 h at room temperature. The slides were mounted with DAPI mounting medium (H-1200, Vector Lab, Burlingame, CA, USA) and photographed under a confocal laser scanning microscope (SP8, Leica, Wetzlar, Germany).

### Cell culture

Primary human corneal epithelial cells (hCECs) were isolated from research-grade human donor corneas (Male, age = 60.5 ± 5.2 years, Eye Bank Association of America, Washington, DC, USA). The use of human donor corneas complied with all relevant ethical regulations and was approved by the Mass General Brigham/Mass Eye and Ear Institutional Biosafety Committee. Procurement of these corneas was performed by the Eye Bank with written consents from donors. hCECs were cultured in Corneal Epithelial Cell Complete Medium (ATCC, Manassas, VA, USA). Human corneal fibroblast cells (hCFCs) were from ATCC and cultured in DMEM/F12 basal medium (Thermo Fisher Scientific, Waltham, MA, USA) containing 10% (v/v) FBS, 1% (v/v) antibiotic/antimycotics (Sigma-Aldrich), and 1% (v/v) l-glutamine (Sigma-Aldrich). The human corneal endothelial cell line (hCEnC-21T) was kindly provided by Dr. Ula Jurkunas (Schepens Eye Research Institute) and cultured in a supplemented Chen medium (OptiMEM-I, Invitrogen). All cells were cultured at 37 °C in a 5% CO_2_ incubator. To determine the biocompatibility of SSOE with cultured cells, hCECs were grown to 100% confluence, and the completed media was replaced with equal volume of SSOE, PBS, or vehicle control for 1 h. hCFCs and hCEnCs were grown to confluence and 2 ml of SSOE, PBS, or vehicle control was placed in a transwell insert, which was then placed in the culture dish for 1 h.

### Cell live/dead staining

Cell viability was evaluated by using LIVE/DEAD™ Viability/Cytotoxicity Kit (L3224, Invitrogen). Briefly, cells were incubated for 30 min at room temperature with a mixture of 2 μM calcein acetoxymethyl ester (Calcein AM) and 4 μM ethidium homodimer-1 (EthD-1). After one-time washing with 1× PBS containing 1 μg/ml Hoechst 33342 (H3570, Invitrogen) to visualize the nucleus, the live cells (green fluorescence) and dead cells (red fluorescence) were photographed using a fluorescence microscope (DMi8, Leica).

### TUNEL staining

In situ cell death was determined by TUNEL (terminal deoxynucleotidyl transferase dUTP nick-end labeling) assay (Roche, Basel, Switzerland) in frozen sections of whole eyes. Sections were counterstained with DAPI and images were taken with the confocal laser scanning microscope (SP8, Leica). DAPI signal (blue) was overlaid with Texas red (TUNEL-positive cells) and quantified using ImageJ software (Version 1.52t). TUNEL positivity was calculated as the ratio of TUNEL-positive cells/DAPI-positive cells (%). For each group, at least three individual mice were included.

### Hematoxylin and eosin staining

Whole eyes were harvested and fixed in 4% paraformaldehyde overnight. In all, 6-μm thick sections were stained with hematoxylin and eosin and examined under a light microscope (DMiL, Leica). The microscopic structures of the anterior and posterior segments of the eye were evaluated, including tissue integrity, structure, and cellular infiltration. The loss of lens transparency or the type of cataract, however, cannot be diagnosed histologically with reliability, as histologic stains that are used to colorize the lens after it is processed prevent the assessment of lens clarity and nuclear and cortical cataractous changes take on a homogeneous eosinophilic appearance.

### RNA isolation and quantitative real-time RT-PCR analysis

Corneal and iris/ciliary body tissues were harvested under dissecting microscope and placed in TRIzol solution (15596026, Invitrogen). Total RNA was isolated with Direct-zol RNA Kit (R2060, Zymo Research, Irvine, CA, USA) following the manufacturer’s protocol and reverse-transcribe to cDNA with RevertAid H Minus Reverse Transcriptase (EP0452, Thermo Fisher Scientific). Real-time PCR was performed using a SYBR Green Master Mix Kit (A25742, Thermo Fisher Scientific) for IL-1β, MMP9, and β-actin, and Taqman Universal PCR Master Mix (Applied Biosystems, Carlsbad, CA, USA) for Cxcl1 and IL-6 on a StepOne plus Real-Time PCR System (Applied Biosystems, Foster City, CA, USA). The cycling conditions for reactions are 40 cycles of 95 °C for 15 s, 60 °C for 60 s. The mouse IL-1β, MMP9 and β-actin gene primers were designed using the Primer 3 system, and their sequences are shown as follows: *β-actin*, 5-cctaaggccaaccgtgaaaag-3, 5-aggcatacagggacagcacag-3; *IL-1β*, 5- tggaccttccaggatgaggaca-3, 5-gttcatctcggagcctgtagtg-3; *MMP9*, 5- gctgactacgataaggacggca-3, 5-tagtggtgcaggcagagtagga-3. The primers for Cxcl1 and IL-6 were Mm04207460_m1 and Mm00446190_m1, respectively. The results of the relative qRT-PCR were analyzed by the comparative threshold cycle (CT) method and normalized to β-actin (IL-1β, MMP9, and β-actin) or GAPDH (Cxcl1 and IL-6) expression as the reference gene.

### Flow cytometric analysis

The conjunctival tissue was separated and subsequently digested in DMEM media containing 2 mg/mL collagenase D (11088866001, Roche, Indianapolis, IN, USA) and 0.2 mg/mL DNase I (10104159001, Roche) for 2 h at 37 °C. The cornea and iris were isolated in RPMI with 2.5 mg/ml Liberase TL (Sigma-Aldrich, St.Louis, MO, USA) and incubated at 37 °C on a shaker for an hour. Tissue suspension was filtered through a 70-µm cell strainer (BD Falcon; Becton-Dickinson, Franklin Lakes, NJ, USA). After washing with 5% FBS, single cells in suspension were incubated with Fc blocking antibody at 4 °C for 30 min. Cells were then immunostained with the APC/Cy7-conjugated anti-CD45 antibody (1:100 dilution, 103116, BioLegend), or isotype-matched control antibody (1:100 dilution, 400624, BioLegend). The Foxp3/transcription factor staining buffer set (00-5523-00, eBioscience), HIF-1α (1:200 dilution, IC1935G, R&D systems, Minneapolis, MN) or isotype-matched control antibody (1:100 dilution, 400132, BioLegend) were used for HIF-1^α^ staining. Live and dead cells were stained using Zombie Green Fixable viability kit (423111, BioLegend). The stained cells were analyzed using the LSRII flow cytometer (BD Biosciences, San Jose, CA, USA) and FCS Express software (De Novo Software, Los Angeles, CA, USA). The gating strategy is described in Supplementary Fig. S5.

### Statistical analysis

The statistical analysis was conducted using GraphPad Prism software version 9.4.1 (458) (GraphPad Software Inc., San Diego, CA, USA). All summary data are reported as means ± SEM. Comparisons between two groups were performed by an unpaired, two-tailed Student’s *t* test; and comparison among three groups were performed by the one-way ANOVA test. The rate of cataract formation between groups was compared by Fisher’s exact test. *P* < 0.05 was considered statistically significant.

### Reporting summary

Further information on research design is available in the Nature Portfolio Reporting Summary linked to this article.

## Supplementary information

Supplementary Information

Reporting Summary

## Data Availability

The data that support this study are available from the corresponding author upon request. The datasets generated during and/or analyzed during this study are available in the Figshare repository, https://figshare.com/s/af23759646b3ad84be8e. Source data are provided with this paper.

## Source data

Source Data
