# Supplementary material for: Perfluorodecalin-based oxygenated emulsion as a topical treatment for chemical burn to the eye
Source: Nat Commun. 2022 Nov 30;13:7371. doi: 10.1038/s41467-022-35241-1 (PMC9712419; doi:10.1038/s41467-022-35241-1)
Supplement: Supplementary file 1 — Supplementary Information [file 41467_2022_35241_MOESM1_ESM.pdf]

SUPPLEMENTARY MATERIALS:

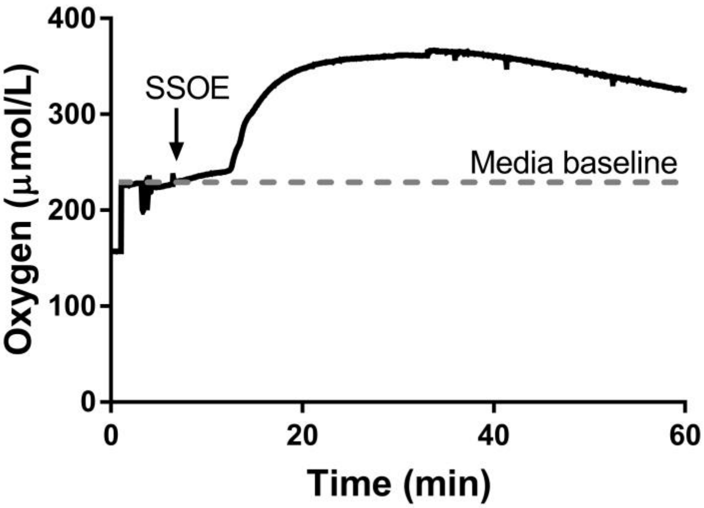

Figure S1. Representative oxygen concentration curve after adding SSOE to cell culture media. Dashed line represents baseline level in culture media alone. Source data are provided as a Source Data file.

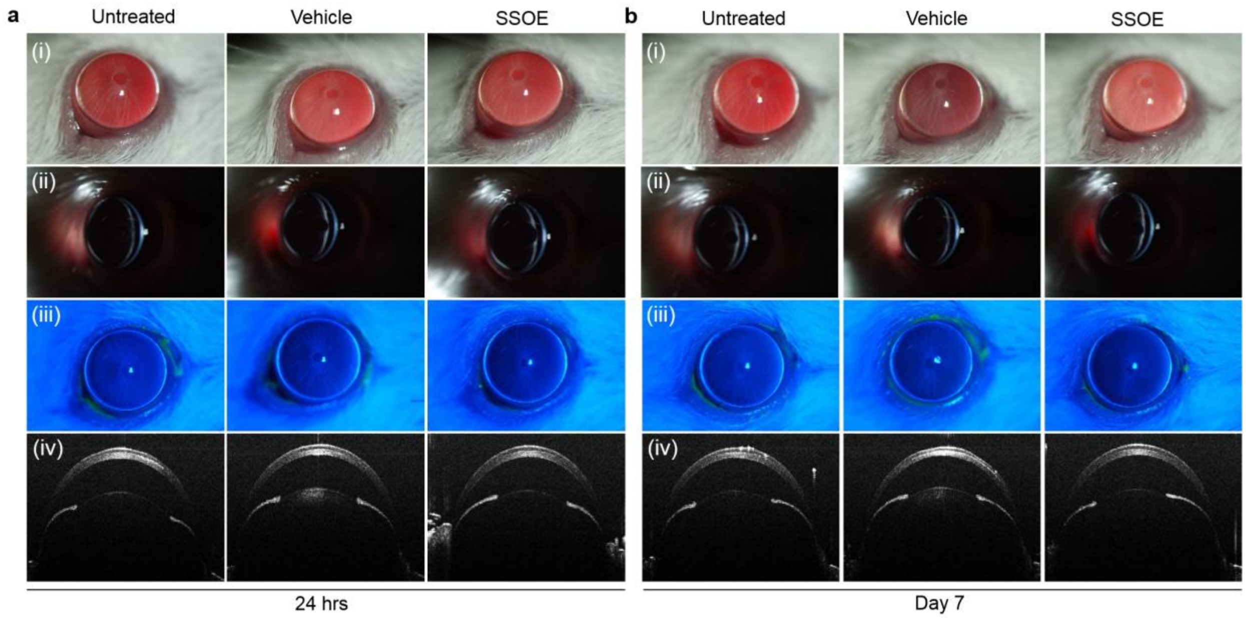

Figure S2. No acute or sub-acute ocular toxicity was observed after SSOE application. SSOE or vehicle were applied to the intact ocular surface of mice for 1 hour, followed by irrigation. Representative slit lamp photography showing no signs of ocular redness, irritation, or inflammation 24 hours (**a**) and 7 days (**b**) after application. (i) Broad beam lighting, (ii) slit beam lighting, (iii) fluorescein staining, and (iv) anterior segment-OCT. Data are representative of three independent experiments.

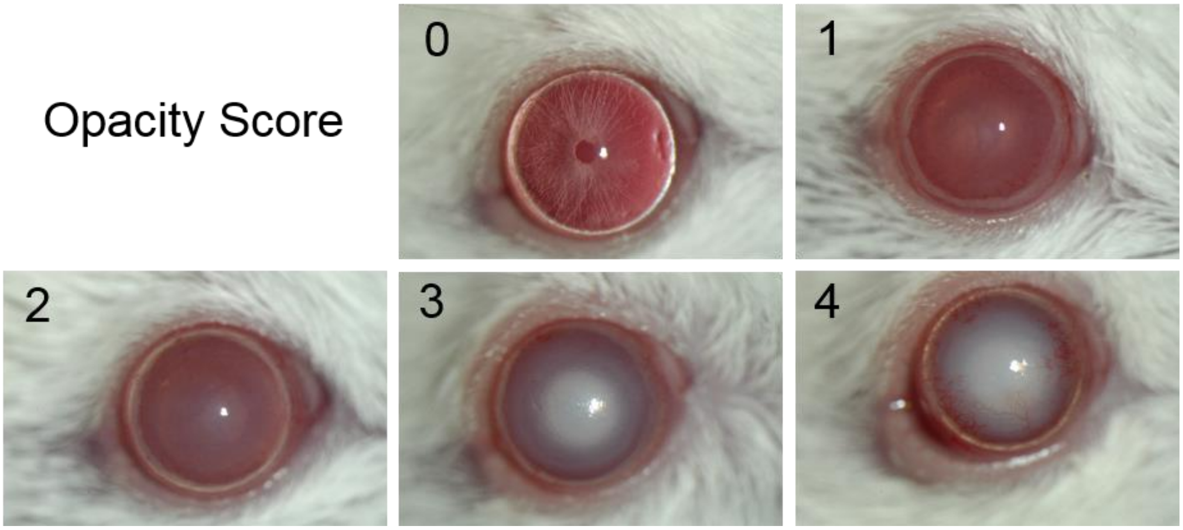

Figure S3. The optical opacity was scored 0 to 4 based on these standard photographs.

Growth factor	Untreated	SSOE	P value
Amphiregulin	0.114	0.109	0.812
bFGF	1.558	1.342	0.333
beta-NGF	0.273	0.282	0.889
EGF	0.788	0.339	0.291
EGFR	0.601	0.460	0.246
GCSF	0.117	0.122	0.885
GM-CSF	0.402	0.415	0.891
FGF-7	0.529	0.575	0.756
HGF	0.026	0.017	0.092
HGFR	0.440	0.471	0.710
IGFBP-2	1.744	1.609	0.522
IGFBP-3	1.765	1.629	0.690
IGFBP-5	0.335	0.322	0.850
IGFBP-6	1.003	0.799	0.202
IGF-1	1.724	1.660	0.848
IGF-1R	0.074	0.027	0.0387*
IGF-2	0.520	0.561	0.765
IL-2	0.251	0.227	0.718
IL-7	0.078	0.059	0.534
M-CSF	0.935	0.942	0.978
PDGF-AA	0.603	0.739	0.421
PDGF-BB	0.186	0.238	0.294
PLGF	0.712	0.579	0.376
SCF	0.114	0.117	0.916
TGF btea1	0.925	0.890	0.843
VEGF-A	0.210	0.192	0.736
VEGFR1	0.177	0.194	0.707
VEGFR2	0.283	0.301	0.847
VEGFR3	0.299	0.350	0.706
VEGF-D	0.258	0.307	0.581

Figure S4. Expression of growth factors in the anterior segment of the eye after SSOE treatment. Mouse cornea, conjunctiva, and iris tissues were collected 7 days post-burn and subjected to growth factor array analysis according to the manufacturer’s protocol (RayBiotech, Peachtree Corners, GA). While the levels of insulin-like growth factor type 1 receptor (IGF-1R, marked with *) was significantly decreased by SSOE treatment, all other growth factors tested showed no significant changes. n = 3 eyes in each group. Statistical significance was determined using unpaired, two-tailed Student’s t-test.

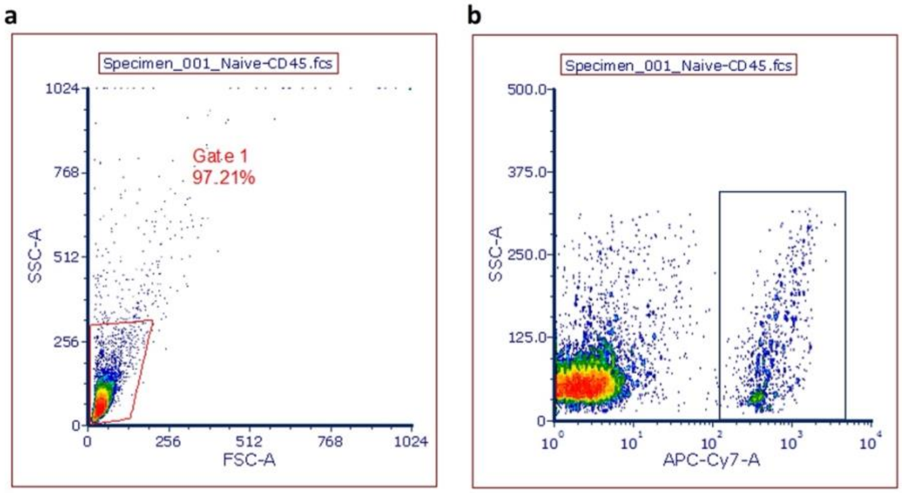

Figure S5. Gating strategies for flow cytometry data analysis. a SSC vs FCS density plot. Forward (FSC-A) and side scatter (SSC-A) were adjusted to minimize events on the axes. More than 90% of total cells were recruited for further gating of the population of CD45 positive cells (**b**).
