# Supplementary material for: Perfluorodecalin-based oxygenated emulsion as a topical treatment for chemical burn to the eye
Source: Nat Commun. 2022 Nov 30;13:7371. doi: 10.1038/s41467-022-35241-1 (PMC9712419; doi:10.1038/s41467-022-35241-1)
Supplement: Supplementary file 2 — Reporting Summary [file 41467_2022_35241_MOESM2_ESM.pdf]

nature portfolio

Corresponding author(s): Jia Yin

Last updated by author(s): 2022 - 11 - 11

Reporting Summary

Nature Portfolio wishes to improve the reproducibility of the work that we publish. This form provides structure for consistency and transparency in reporting. For further information on Nature Portfolio policies, see our Editorial Policies and the Editorial Policy Checklist.

Statistics

For all statistical analyses, confirm that the following items are present in the figure legend, table legend, main text, or Methods section.

n/a	Confirmed	
[ ]	[x]	The exact sample size (*n*) for each experimental group/condition, given as a discrete number and unit of measurement
[ ]	[x]	A statement on whether measurements were taken from distinct samples or whether the same sample was measured repeatedly
[ ]	[x]	The statistical test(s) used AND whether they are one- or two-sided<br>*Only common tests should be described solely by name; describe more complex techniques in the Methods section.*
[x]	[ ]	A description of all covariates tested
[ ]	[x]	A description of any assumptions or corrections, such as tests of normality and adjustment for multiple comparisons
[x]	[ ]	A full description of the statistical parameters including central tendency (e.g. means) or other basic estimates (e.g. regression coefficient) AND variation (e.g. standard deviation) or associated estimates of uncertainty (e.g. confidence intervals)
[x]	[ ]	For null hypothesis testing, the test statistic (e.g. *F*, *t*, *r*) with confidence intervals, effect sizes, degrees of freedom and *P* value noted<br>*Give P values as exact values whenever suitable.*
[x]	[ ]	For Bayesian analysis, information on the choice of priors and Markov chain Monte Carlo settings
[x]	[ ]	For hierarchical and complex designs, identification of the appropriate level for tests and full reporting of outcomes
[x]	[ ]	Estimates of effect sizes (e.g. Cohen's *d*, Pearson's *r*), indicating how they were calculated

Our web collection on statistics for biologists contains articles on many of the points above.

Software and code

Policy information about availability of computer code

Data collection	No software was used for data collection.
Data analysis	Data analysis was done using GraphPad Prism software 9.4.1 (458) and ImageJ software Version 1.52t.

For manuscripts utilizing custom algorithms or software that are central to the research but not yet described in published literature, software must be made available to editors and reviewers. We strongly encourage code deposition in a community repository (e.g. GitHub). See the Nature Portfolio guidelines for submitting code & software for further information.

Data

Policy information about availability of data

All manuscripts must include a data availability statement. This statement should provide the following information, where applicable:

- Accession codes, unique identifiers, or web links for publicly available datasets
- A description of any restrictions on data availability
- For clinical datasets or third party data, please ensure that the statement adheres to our policy

The datasets generated during and/or analyzed during the current study are available in the Figshare repository, https://figshare.com/s/af23759646b3ad84be8e. Source data are provided with this paper.

Human research participants

Policy information about studies involving human research participants and Sex and Gender in Research.

Reporting on sex and gender	*Use the terms sex (biological attribute) and gender (shaped by social and cultural circumstances) carefully in order to avoid confusing both terms. Indicate if findings apply to only one sex or gender; describe whether sex and gender were considered in study design whether sex and/or gender was determined based on self-reporting or assigned and methods used. Provide in the source data disaggregated sex and gender data where this information has been collected, and consent has been obtained for sharing of individual-level data; provide overall numbers in this Reporting Summary. Please state if this information has not been collected. Report sex- and gender-based analyses where performed, justify reasons for lack of sex- and gender-based analysis.*
Population characteristics	*Describe the covariate-relevant population characteristics of the human research participants (e.g. age, genotypic information, past and current diagnosis and treatment categories). If you filled out the behavioural & social sciences study design questions and have nothing to add here, write "See above."*
Recruitment	*Describe how participants were recruited. Outline any potential self-selection bias or other biases that may be present and how these are likely to impact results.*
Ethics oversight	*Identify the organization(s) that approved the study protocol.*

Note that full information on the approval of the study protocol must also be provided in the manuscript.

Field-specific reporting

Please select the one below that is the best fit for your research. If you are not sure, read the appropriate sections before making your selection.

☒ Life sciences ☐ Behavioural & social sciences ☐ Ecological, evolutionary & environmental sciences

For a reference copy of the document with all sections, see nature.com/documents/nr-reporting-summary-flat.pdf

Life sciences study design

All studies must disclose on these points even when the disclosure is negative.

Sample size	Based on our preliminary of optic opacity score, the mean values of control and SSOE groups are 3.54 and 1.67 respectively, the standard deviation is 0.99. A two-sided P value of 0.05 and a power of 0.8 will require 9 mice per group. For in vitro testing, a minimum sample size of 3 in each group was determined to adequately perform statistical analysis.
Data exclusions	No data points were excluded.
Replication	All experiments were repeated at least twice and all attempts at replication were successful.
Randomization	*Describe how samples/organisms/participants were allocated into experimental groups. If allocation was not random, describe how covariates were controlled OR if this is not relevant to your study, explain why.*
Blinding	Clinical assessment of animal experiments was performed by a masked observer.

Reporting for specific materials, systems and methods

We require information from authors about some types of materials, experimental systems and methods used in many studies. Here, indicate whether each material, system or method listed is relevant to your study. If you are not sure if a list item applies to your research, read the appropriate section before selecting a response.

Materials & experimental systems

n/a	Involved in the study	
☐	☒	Antibodies
☐	☒	Eukaryotic cell lines
☒	☐	Palaeontology and archaeology
☐	☒	Animals and other organisms
☒	☐	Clinical data
☒	☐	Dual use research of concern

Methods

n/a	Involved in the study	
☒	☐	ChIP-seq
☐	☒	Flow cytometry
☒	☐	MRI-based neuroimaging

Antibodies

Antibodies used	alpha-SMA antibody: eBioscience, catalog# 14-6496-82, Clone HHF35<br>CD45 antibody : Biolegend, catalog# 103101, Clone 30-F11<br>Alexa Fluor 488-conjugated donkey anti-mouse secondary antibody: Invitrogen, Catalog#A-21202<br>Alexa Fluor 488-conjugated donkey anti-rat secondary antibody: Invitrogen, Catalog#A-21208<br>APC/Cy7-conjugated anti-CD45 antibody: BioLegend, catalog# 103116, Clone 30-F11<br>APC/Cyanine7 Rat IgG2b, κ Isotype Ctrl Antibody: Biolegend, catalog#400624, Clone RTK4530<br>HIF-1 alpha Alexa Fluor 488-conjugated Antibody: R&D systems, catalog# IC1935G, Clone # 241812<br>Alexa Fluor® 488 Mouse IgG1, κ Isotype Ctrl (FC) Antibody: Biolegend, catalog#400132, clone MOPC-21.
Validation	alpha-SMA antibody: eBioscience, catalog# 14-6496-82, Clone HHF35. Monoclonal antibody recognizes muscle actin; specifically the alpha and gamma form in human, mouse, rat, bovine, pig, guinea pig, sheep, frog, canine and non-human primate. It has been tested in immunohistochemistry by the manufacturer and used in published studies. Host/Isotype - Mouse/IgG1, kappa.<br><br>CD45 antibody: Biolegend, catalog# 103101, Clone 30-F11. Monoclonal antibody recognizes mouse CD45 and has been verified in immunohistochemistry by the manufacturer. Host/Isotype - Rat IgG2b, κ<br><br>Alexa Fluor 488-conjugated donkey anti-mouse secondary antibody: Invitrogen, Catalog#A-21202. Polyclonal secondary antibody with gamma immunoglobins heavy and light chains as immunogen. This has been verified in immunohistochemistry by the manufacturer. Host/isotype - Donkey/IgG.<br><br>Alexa Fluor 488-conjugated donkey anti-rat secondary antibody: Invitrogen, Catalog#A-21208. Polyclonal secondary antibody with gamma immunoglobins heavy and light chains as immunogen. This has been verified in immunohistochemistry by the manufacturer. Host/isotype - Donkey/IgG.<br><br>APC/Cy7-conjugated anti-CD45 antibody: BioLegend, catalog# 103116, Clone 30-F11. Monoclonal antibody recognizes mouse CD45. It has been quality-tested in flow cytometry by the manufacturer. Host/Isotype - Rat IgG2b, κ<br><br>APC/Cyanine7 Rat IgG2b, κ Isotype Ctrl Antibody: Biolegend, catalog#400624, Clone RTK4530. Monoclonal antibody was chosen as an isotype control after screening on a variety of resting, activated, live, and fixed mouse, rat and human tissues and has been quality-tested in flow cytometry by the manufacturer. Host/Isotype - Rat IgG2b, κ.<br><br>HIF-1 alpha Alexa Fluor 488-conjugated Antibody: R&D systems, catalog# IC1935G, Clone # 241812. Monoclonal antibody recognizes human and mouse HIF-1 alpha. It has been tested in flow cytometry by the manufacturer. Host/Isotype - Mouse IgG1.<br><br>Alexa Fluor® 488 Mouse IgG1, κ Isotype Ctrl (FC) Antibody: Biolegend, catalog#400132, clone MOPC-21. The isotype of this monoclonal antibody is mouse IgG1, κ. This antibody was chosen as an isotype control after screening on a variety of resting, activated, live, and fixed mouse, rat and human tissues. It has been tested in flow cytometry by the manufacturer. Host/Isotype - Mouse IgG1, k.

Eukaryotic cell lines

Policy information about cell lines and Sex and Gender in Research

Cell line source(s)	Human corneal endothelial cell line hCEnC-21T was kindly provided by Dr. Ula Jurkunas of the Schepens Eye Research Institute of Mass Eye and Ear. The cell line was derived from a 21-year-old male donor and transduction with telomerase yielded this highly proliferative cell line.<br><br>Primary human corneal epithelial cells were isolated from research grade human donor corneas (Eye Bank Association of America, Washington, DC). The use of human donor corneas complied with all relevant ethical regulations and was approved by the Mass General Brigham/Mass Eye and Ear Institutional Biosafety Committee. Procurement of these corneas was performed by the Eye Bank with written consents from donors. The donors were male with mean age of 60.5 ± 5.2 years.
Authentication	Human corneal endothelial cell line hCEnC-21T: In the original publication by Dr. Jurkunas (PMID: 23284695), the cell line was authenticated to be devoid of oncogenic transformation and retain critical corneal endothelial cell characteristics and functionality. The cell line has since been used in multiple studies and reported in the literature.<br><br>Primary human corneal epithelial cells: In preliminary study, these cells were immunostained with keratin 12, a marker for mature corneal epithelial cells.
Mycoplasma contamination	The cell line and primary cells were not tested for mycoplasma contamination.
Commonly misidentified lines<br>(See ICLAC register)	*Name any commonly misidentified cell lines used in the study and provide a rationale for their use.*

Animals and other research organisms

Policy information about studies involving animals; ARRIVE guidelines recommended for reporting animal research, and Sex and Gender in Research

Laboratory animals	BALB/C mice, male, age 8-10 weeks, were obtained from the Charles River Laboratories. The mice were kept at a 10/14-hour dark/light cycle with ambient temperature 21-23°C, and humidity 40-60%.
Wild animals	The study did not involve wild animals.
Reporting on sex	Sex was considered a biological variable in the study. Preliminary study used equal numbers of male and female adult mice and showed no sex-based differences in their response to alkali burn or SSOE treatment. In the current study, adult male mice between the ages of 8 and 10 weeks were used.
Field-collected samples	The study did not involve samples collected from the field.
Ethics oversight	Animal Care Committee of the Schepens Eye Research Institute

Note that full information on the approval of the study protocol must also be provided in the manuscript.

Flow Cytometry

Plots

Confirm that:

- [x] The axis labels state the marker and fluorochrome used (e.g. CD4-FITC).
- [x] The axis scales are clearly visible. Include numbers along axes only for bottom left plot of group (a 'group' is an analysis of identical markers).
- [x] All plots are contour plots with outliers or pseudocolor plots.
- [x] A numerical value for number of cells or percentage (with statistics) is provided.

Methodology

Sample preparation	Cornea, conjunctiva, and iris tissues were collected in media containing collagenase D or Liberase TL.
Instrument	LSRII flow cytometer (BD Biosciences, San Jose, CA)
Software	FCS Express software (De Novo Software, Los Angeles, CA).
Cell population abundance	Cell population and purity were determined post-sort with negative controls.
Gating strategy	Gating strategies were confirmed in preliminary experiments.

- [x] Tick this box to confirm that a figure exemplifying the gating strategy is provided in the Supplementary Information.
